# The prevalence of self-injury in adolescence: a systematic review and meta-analysis

**DOI:** 10.1007/s00787-023-02264-y

**Published:** 2023-07-24

**Authors:** Bernadett Frida Farkas, Zsofia K. Takacs, Nóra Kollárovics, Judit Balázs

**Affiliations:** 1 Mental Health Sciences Doctoral School, Semmelweis University, Budapest, Hungary; 2 Faculty of Education and Psychology, Institute of Education, Eötvös Loránd University, Budapest, Hungary; 3 School of Health in Social Sciences, University of Edinburgh, Edinburgh, UK; 4 Department of Developmental and Clinical Child Psychology, Faculty of Education and Psychology, Institute of Psychology, Eötvös Loránd University, Budapest, Hungary; 5 Oslo New University College, Oslo, Norway

**Keywords:** Adolescents, Self-injurious behavior, Prevalence, Systematic review, Meta-analysis

## Abstract

In the past 10 years, there has been a growing interest in self-injurious behavior (SIB) among adolescents. The lifetime prevalence of SIB is between 16 and 22% in community sample with females more likely to engage in SIB. There are conflicting results about the global distribution of the prevalence of SIB and whether the SIB has increased in the 21st century. Our aim in the current study was to conduct a systematic search of and meta-analysis on the prevalence of SIB in adolescents over the past 5 years’ worth of published papers and to examine gender, continental, and year differences. We conducted a systematic search in June 2020 of six databases (PubMed, Scopus, Web of Science, OVID Medline, PsycINFO, EBSCO) with three main search terms: “self-injurious behavior,” “prevalence,” and “adolescence.” Article inclusion criteria were (a) written in English; (b) published between January 1, 2015, and June 18, 2020; and (c) focused on a community sample. Titles and abstracts of the articles were screened first. Then, the relevant full texts were read, and those that met the inclusion criteria were collected. We used Comprehensive Meta-Analysis software was used to conduct the analyses. After the screening process 97, articles were included in the meta-analysis. The age of the samples ranged from 11.00 to 18.53 years. The overall average prevalence of nonsuicidal self-injury in the studies was 16%. There was a significant gender difference: females reported a higher prevalence than males (19.4% and 12.9%, respectively). A significantly higher prevalence was found among Asian articles than those from other continents (19.5% and 14.7%, respectively). The prevalence of SIB did not change significantly between 2013 and 2018. The current research draws attention to the high prevalence of SIB among adolescents, especially among females and those living in Asia. It is important to address this behavior, both in terms of prevention and intervention.

**Supplementary Information:**

The online version contains supplementary material available at 10.1007/s00787-023-02264-y.

## Background

Self-injurious behavior (SIB) is a phenomenon whereby a person directly and deliberately damage themselves [1]. Various terms are used in the literature, such as, *nonsuicidal self-injury* (NSSI), *deliberate self-harm* (DSH), *self-cutting*, and *self-harm* [2–6]. SIB can be considered as a class of behaviors on a spectrum from NSSI—in which the person has no intent to die—to suicidal attempt (SA), which is a form of suicidal behavior [1]. Although NSSI and SA are two distinct behaviors, it is not always easy to decide whether there was a suicidal intent behind SIB, and in this case, it is difficult to separate them [1, 3]. Moreover, the comorbidity between NSSI and suicidal behaviors is approximately 50% in traditional and 70% in clinical populations [1, 7, 8]. Previous research has indicated that the higher risk of SA is associated with the following in regard to NSSI: greater frequency, more methods, and longer duration [1].

Several meta-analyses conducted in the past 20 years have focused on the prevalence of SIB [6, 9–12]; see Table 1).

**Table 1:** Previous meta-analyses

Author	Year of publication	Number of articles	Age range	Continental differences	Main results
Muehlenkamp et al. [10]	2012	52	11–24	–	Lifetime prevalence: 18% NSSI, 16.1% DSH 12-months prevalence: 19% NSSI Average lifetime prevalence did not change between 2005 and 2011
Swannell et al. [9]	2014	34	Adults: 25< Young adults: 18–24 Adolescents: 10–17	No significant difference	1990–1999: 11.7% 2000–2005: 14.7% 2006–2012: 19.3% Pooled lifetime prevalence: 19.7% Females NSSI: 19.9% Males NSSI: 14.7%
Bresin and Schoenleber [11]	2015	116	11.55–55.5 (M = 20.81)	–	Females NSSI: 33.78% Males NSSI: 26.36%
Gillies et al. [6]	2018	172	12–18 (M = 12.81)	Difference duo to methodological factors	Lifetime prevalence: 16.9% (DSH—11.4%, NSSI—22.9%) Significant increasing over time Girls were more likely to self-harm
Lim et al. [12]	2019	66	12.59–19.78 (mean age)	Lifetime prevalence of NSSI: higher in non-Western countries (32.6%) vs. Western (19.4%) Lifetime prevalence of DSH: higher in Western countries (14.2%) vs. non-Western (12.8%) Lifetime prevalence of NSSI was higher among low- and middle-income countries vs. developed Lifetime prevalence of NSSI was highest in Australia (30.9%) lowest in Europe (18.4%) Lifetime prevalence of DSH was highest in Asia (17.4%) and lowest in North America (7.3%)	Lifetime prevalence: 22.1% NSSI, 13.7% DSH 12-months prevalence: 19.5% NSSI, 14.2% DSH

*NSSI* nonsuicidal self-injury, *DSH* deliberate self-harm, *M* mean age

Based on the previous meta-analyses, the prevalence of SIB shows a significant increase in the 1990s, but then a stagnation from 2005 [9, 10]. Gillies et al. (2018) found that the lifetime prevalence increased over time from 1990 to 2015, and Muehlenkamp et al. (2012) found no significant difference in the prevalence of NSSI and DSH between 2005 and 2011 [6, 10]. According to previous meta-analyses, between 1990 and 2015, the average lifetime prevalence of SIB among adolescents is between 16.9 and 19.7% [6, 9–12].

There are conflicting results about the gender differences in the prevalence of SIB [6, 9–12]. Some research has indicated that females have a lifetime prevalence of SIB that is two to three times higher than males [11], whereas other studies have found that the gender difference does not appear at all or, if it does, to a lesser extent [9, 10].

As shown in Table 1 the results are conflicting in regard to whether there is a difference in the prevalence of NSSI and DSH [6, 10, 12]. Muehlenkamp et al. (2012) did not find any significant difference between the two phenomena, however Gillies et al. (2018) and Lim et al. (2019) found a significantly higher prevalence of NSSI than DSH [6, 10, 12]. The lowest lifetime prevalence of NSSI was 18%, while the highest was 22.9% [6, 9–12]. At the same time, the lowest lifetime prevalence of DSH was 11.4%, while the highest was 16.1% [6, 9–12]. As mentioned above, unlike NSSI, DSH can be suicidal and nonsuicidal as well, but it must be a non-fatal self-harm [6, 12].

There are also conflicting results about the continental differences in the prevalence of SIB. While Swannell et al. (2014) didn’t find any significant continental differences in the prevalence of SIB, Gillies et al. (2018) did: Sweden had the highest, Norway had the lowest prevalence of self-harm in the meta-analysis [6, 9]. Lim et al. found that the lifetime prevalence of both NSSI and DSH were higher in non-Western countries than Western countries [12].

The above described previous meta-analyses highlight that these conflicting results can be due to the large differences among the included studies in methodological factors (e.g., sampling method, measurement, mean age of the sample), in the definitions of SIB (e.g., with or without suicidal intent), or in the place of data collection [6, 9–12].

## Aims

Because of the heretofore conflicting results, our aim in this study was to follow up previous meta-analyses on the prevalence of SIB in adolescent community samples [6, 11]. We focused on data published between 2015 and 2020.

Our first hypothesis was that the prevalence of SIB did not change over time between the examined period (2015 and 2020) for both females and males. Our second hypothesis was that females reported a higher prevalence of a history of SIB than males.

Before addressing these hypotheses, we investigated the following questions: (a) What kinds of definitions of SIB are used? (b) What kinds of assessments are used to measure SIB? (c) What was the sampling method? (d) Where were the data of the included studies collected? (e) What was the mean age of the sample? (f) Was suicidal intent excluded from the definition of SIB?

## Method

### Literature search

We conducted a systematic literature search on June 18, 2020. We used six computerized literature databases: PubMed, Scopus, Web of Science, OVID Medline, PsycINFO, and EBSCO Discovery Service for Semmelweis University. Search terms were the following: “non-suicidal” OR nonsuicidal OR “self-injur*” OR “self-harm” OR “self harm” OR parasuic* OR “self-mutilation” AND prevalence AND adolescen* NOT “clinical trial” OR “case report” OR review. Two filters were added: (a) date between January 1, 2015, and June 18, 2020, and (b) English language. We used EndNote X9 software to remove duplicates and screen the 374 search hits.

### Inclusion and exclusion criteria

To be included, studies had to report on the prevalence of SIB in adolescence in a community sample in a published article written in English. We used “adolescen*” among our search terms, and we included studies with an age range 11–18 years. However, in some articles this age range was wider (22 years being the oldest), so from these we included in the final analysis only those studies where the mean age of the sample was adolescence. For details, see Table 2.

**Table 2:** The included relevant studies

Author (year)	Country	Year of data collection	Mean age (SD)	Sample size (% female)	Sampling	Terminology	Suicidal intent distinction	Measurement	Prevalence
Aldrich et al. (2018) [28]	USA	2013–2015	12.86 (0.85)	121 (55.4%)	C	SITB	No	Questionnaires validated for other than SIB	Lifetime: 18.20% overall 6-months: 16.1% female, 12% male
Badoud et al. (2015) [29]	Switzerland	–	15.72 (1.74)	130 (50.8%)	C	NSSI	Yes	Questionnaires validated for other than SIB	1-year: 25.4% overall 24.24% female 26.6% male
Baetens et al. (2015) [30]	Belgium	2011	16.07 (1.12)	358 (48%)	C	NSSI	Yes	Questionnaires validated for other than SIB	1-year: 9.78%; lifetime: 13.41%
Barrocas et al. (2015) [31]	China	–	16.02 (0.61)	617 (51.4%)	C	NSSI	Yes	Not validate	Lifetime: 23.8% overall 26% male 21.7% female
Bhola et al. (2017) [32]	India	2013	17.5 (14.2)	1571 (57.8%)	RA	NSSI	Yes	Validate for SIB	1-year: 33.8%
Brausch and Woods (2019) [33]	USA	–	13.19 (1.19)	436 (52.7%)	C	NSSI	Yes	Validate for SIB	6-months: 17.2% overall 14.62% male 18.97% female
Buelens et al. (2020) [34]	Belgium	2018	15 (1.81)	2130 (54%)	C	NSSI/NSSI-D	Yes	Single-item question	Lifetime: 21.8% → 7.6% met the NSSI-D diagnosis 29.9% female, 12% male; NSSI-D diagnosis: 11.7% female, 2.9% male
Calvete et al. (2015) [35]	Spain	2010	15.32 (1.97)	1864 (51.45%)	RA	NSSI	Yes	Validate for SIB	1-year: 55.6% overall 58% female 53.3% male
Carvalho et al. (2017) [36]	Portugal	2012	16.75 (1.31)	1763 (52.9%)	C	NSSI	Yes	Validate for SIB	Lifetime: 29.5% overall 29.4% male 29.7% female
Cassels et al. (2018) [37]	UK	2005–2008	–	1238 (54.5%)	C	NSSI	Yes	Questionnaires validated for other than SIB	Lifetime: 11.89%
Castro and Kirchner (2017) [38]	Chile	–	14.98 (1.69)	965 (57%)	C	NSSI	Yes	Validate for SIB	Lifetime: 49.6% overall 41.39% male 55.45% female
Chen and Chun (2019) [39]	Taiwan	2011–2012	15.23 (1.24)	438 (100%)	C	NSSI	Yes	Validate for SIB	1-year: 36.8%
Çimen et al. (2017) [40]	Turkey	2014	15.00 (1.13)	555 (56.6%)	C	NSSI	Yes	Validate for SIB	Lifetime prevalence: 11.4%
Claes et al. (2015) [41]	Belgium, Netherlands	2013	15.56 (1.32)	785 (44.5%)	C	NSSI	Yes	Validate for SIB	Lifetime prevalence: 20.1%
Copeland et al. (2019) [42]	USA	2009–2010	–	5870 (56.6%)	C	Self-cutting	No	Single-item question	1 year: 7% overall 4.67% male 11.35% female
Donath et al. (2019) [43]	Germany	2015	14.91 (0.73)	10,638 (49.8%)	RE	D-SIB	No	Single-item question	1 year: 17.8% overall 5.1% male 29.7% female
Doyle et al. (2015) [16]	Ireland	1999–2000	M = 16 years, SD = 0.715	856 (48.8%)	RE	SH	No	Validate for SIB	Lifetime: 12.1% overall 18.1% female 6.4% male
Duarte et al. (2019) [44]	Portugal	2017–2018	Study 1: 16.1 (1.8) Study 2: 15.4 (1.8)	Study 1: 620 (67.9%) Study 2: 411 (67.9%)	C	DSH	No	Validate for SIB	Lifetime: 21.1% in study 1, 26.5% in study 2
Emerson et al. (2019) [45]	Australia	2000–2001	14	9845 (47.6%)	RE	SH	No	Single-item question	1 year: 14.9%
Emery et al. (2017) [46]	Canada	–	13.38 (0.51)	639 (53%)	C	NSSI	Yes	Validate for SIB	Lifetime: 18% overall 22.7% female 13% male
Endo et al. (2017) [47]	Japan	2008–2009	15.2 (1.7)	17,347 (50.2%)	C	SH	No	Single-item question	1 year: 3.8%
Esposito et al. (2019) [48]	Italy	2016	15.60 (1.65)	640 (60.5%)	C	NSSI	Yes	Not validate	6-months: 15.3% overall 14.6% male 15.8% female
Farhat et al. (2020) [49]	USA	2000	–	2234 (43.9%)	C	SIB	Yes	Single-item question	Lifetime: 18% overall 24% female, 13.7% male
Fraser et al. (2018) [50]	New Zealand	2012–2015	15.16 (2.61)	1799 (56.5%)	C	NSSI	Yes	Validate for SIB	Lifetime: 20.6% overall 28.7% female 9.9% male
Gandhi et al. (2015) [51]	Belgium	2014	16.13 (1.47)	568 (61.8%)	C	NSSI	Yes	Validate for SIB	Lifetime: 16.5% overall 12.8% female 3.9% male
Gandhi et al. (2017) [52]	Belgium	2015–2016	15.0 (1.84)	528 (50.4%)	C	NSSI	Yes	Single-item question	Lifetime:14.2% overall 20.8% female 7.7% male
Gandhi et al. (2018) [53]	Belgium	2012–2013	–	3880 (51%)	C	NSSI	Yes	Single-item question	Lifetime: 21% overall 26% female 17% male
Gandhi et al. (2018) [54]	Belgium	2015	16.6 (0.96)	401 (51.5%)	C	NSSI	Yes	Single-item question	Lifetime: 16.5% overall 20.77% female 11.85% male
Gandhi et al. (2019) [55]	Belgium	2015–2017	15.0 (1.85)	528 (50.4%)	C	NSSI	Yes	Single-item question	1 year: 7.55%
Garisch and Wilson (2015) [56]	New Zealand	2008–2009	16.35 (0.62)	1162 (43%)	RE	NSSI	Yes	Validate for SIB	Lifetime: 48.7% overall 49.4% female 48% male
Gaspar et al. (2019) [57]	Portugal	2014	14.8 (1.2)	3262 (54%)	RE	NSSI	No	Single-item question	1 year: 20.3% overall females 23.7% males 16.3%
Geulayov et al. (2018) [58]	UK	2015	–	5520 (51%)	RE	Non-fatal SH	No	Not validate	1 year: 5.83% overall 8.9% females 2.6% males
Gromatsky et al. (2017) [59]	USA	2013–2014	14.39 (0.63)	550 (100%)	C	NSSI	Yes	Validate for SIB	Lifetime: 7.82%
Guerreiro et al. (2015) [60]	Portugal	2009–2011	15.6 (1.7)	1713 (55.6%)	C	SH	No	Validate for SIB	Lifetime: 7.3% overall 10.5% females 3.3% males
Hamada et al. (2016) [61]	Japan	2011	13.9 (0.2)	1840 (51.4%)	C	Self-cutting	No	Single-item question	Lifetime: 8.9% overall 5.6% males 11.9% females
Han et al. (2018) [62]	China	2013	14.81	5726 (50.3%)	RA	SH	Yes	Not validate	6 months: 45.3% overall 41.6% male 49% female
Hanania et al. (2015) [63]	Jordan	–	14.53 (1.71)	952 (49.8%)	C	NSSI	Yes	Validate for SIB	Lifetime: 22.6% overall males 26.98% females 18.14%
Heerde et al. (2015) [64]	USA, Australia	2002–2003	Washington State: 14.1 Grade 7, 15.1 Grade 9; Victorian: 13.9 Grade 7, 14.9 Grade 9	3876 (51%)	RE	DSH	No	Single-item question	1 year: 1.53% in Grade 7 and 0.91% in Grade 9 for males, 4.12% and 1.34% for Grade 7 and Grade 9 for females
Horváth et al. (2018) [65]	Hungary	2009–2010, 2013	Vocational school sample: 15.21 (0.77) High-school sample: 15.09 (0.75)	Vocational school sample: 140 (40%); high-school sample: 995 (59.2%)	RE, RA	D-SIB	Yes	Validate for SIB	Lifetime: 29.4% in the vocational school group, 17.2% in the high school group Vocational school sample: 25.64% males, 35.41% females High-school sample: 14.4% males, 19% females
Horváth et al. (2020) [66]	Hungary	2015–2017	15.43 (1.14)	161 (50%)	C	NSSI	Yes	Validate for SIB	Lifetime: 23.6% overall 8.64% males 33.75% females
Huang et al. (2017) [15]	Taiwan	2008–2010	16.02 (0.52)	5879 (56.7%)	C	DSH	Yes	Single-item question	Lifetime: 25.04% overall 28.96% female 19.9% male
Jantzer et al. (2015) [67]	Germany	2012	12.8 (1.95)	647 (50.7%)	C	NSSI	Yes	Single-item question	1 year: 10.97%
Jiang et al. (2016) [68]	China	2013–2014	13.17 (1.10)	813 (43.4%)	C	NSSI	Yes	Single-item question	Lifetime: 29.0% overall 27.9% male 31.3% female
Kądziela-Olech et al. (2015) [69]	Poland	2013	16.7 (1.64)	2220 (46.3%)	C	D-SIB, NSSI	Yes	Validate for SIB	D-SIB lifetime: 8.3%; NSSI lifetime: 4.8% D-SIB lifetime: 6.7% females, 9.6% males; NSSI lifetime: 6.3% males, 3.0% females
Kaess et al. (2020) [70]	10 European countries + Israel	2009–2010	14.84 (0.9)	1933 (51.47%)	RE	D-SIB	Yes	Validate for SIB	Lifetime: 24.9%; 1-year: 6.7% overall 7.04% male 6.43% female
Kang et al. (2018) [71]	China	–	15.63 (1.67)	3555 (52.0%)	RA	NSSI	Yes	Validate for SIB	6-months: 13.8% overall 16.6% female 10.4% male
Kelada et al. (2016) [72]	Australia	2014	14.49 (1.38)	117 (56.4%)	C	Self-injury	Yes	Single-item question	Lifetime: 19.7%
Kiekens et al. (2015)[73]	Netherlands, Belgium	2012	15.52 (1.34)	946 (44%)	RA	NSSI	Yes	Validate for SIB	Lifetime: 24.31% overall 24.46% male 24.26% female
Kitagawa et al. (2017) [74]	Japan	2008–2009	–	18,018 (50.3%)	C	SH	Yes	Single-item question	1 year: 7.3%
Klemera et al. (2016) [75]	UK	2013–2014	15	1519 (48.8%)	RE	SH	No	Single-item question	Lifetime: 21.5% overall 31.9% females 11.4% males
Koenig et al. (2016) [76]	Germany	2010–2012	14.7	506 (52.1%)	RE	D-SIB	Yes	Validate for SIB	1-year: 8.30% overall lifetime: 30.7% male 47.45% female
Latina and Stattin (2017) [77]	Sweden	2008, 2010	13.89 (0.75)	2029 (50%)	RE	SH	Yes	Validate for SIB	6 months: 31%
Law and Shek (2016) [78]	China	–	12.53 (0.66)	2023 (52%)	RA	SH	Yes	Not validate	Lifetime: 15.3% overall 13.9% male 16.5% female
Lee (2016) [79]	South Korea	–	14.38 (1.68)	784 (48.8%)	C	SH	No	Validate for SIB	Lifetime: 12.4%
Li et al. (2019) [80]	China	2015–2016	15.36 (1.79)	22,628 (51.4%)	RE	NSSI	No	Not validate	12 months: 32.1% overall male 35.2% female 29.1%
Lin et al. (2017) [81]	Taiwan	2013	15.83 (0.38)	2170 (51.5%)	C	NSSI	Yes	Single-item question	1 year: 20.1% overall female 23.8% male 16.9%
Liu et al. (2017) [82]	Taiwan	2008–2009	15.44 (0.61)	2479 (60.3%)	C	SH	Yes	Single-item question	1 year: 10.1% overall female 11.24% male 8.32%
Liu et al. (2018) [83]	China	2015	14.97 (1.46)	11,831 (49.1%)	RE	NSSI	Yes	Validate for SIB	Lifetime: 23.7%; 1-year: overall 18.9% male 17.8% female 19.9%
Luyckx et al. (2015) [84]	Belgium	–	15.95 (1.30)	348 (100%)	C	NSSI	Yes	Validate for SIB	Lifetime: 20.7%
Lüdtke et al. (2017) [85]	Switzerland	2010	14.95 (0.74)	447 (48%)	C	NSSI	Yes	Validate for SIB	1 year: 5.15% male 13.08% female
Madjar et al. (2019) [86]	Israel	–	14.96 (1.33)	594 (45.6%)	C	NSSI	Yes	Validate for SIB	1-year: 19.5% male, 10.7% female
Mars et al. (2019) [87]	UK	–	16.8 (2.9)	4795 (73%)	RE	NSSI	Yes	Single-item question	Lifetime: 11.73% overall 5.7% male, 15.92% female
Martinez-Ferrer and Stattin (2019) [88]	Sweden	–	13.94 (0.74)	987 (48.3%)	C	SH	Yes	Validate for SIB	6 months: 36%
McManus et al. (2020) [89]	UK	2000, 2007, 2014	–	2000: 103 2014: 122	RE	NSSH	Yes	Single-item question	Lifetime: 6.1% in 2000, 10.9% in 2014
Monto et al. (2018) [90]	USA	2015	–	64 671 (52.5%)	RE	NSSI	Yes	Single-item question	1-year: 23.8% female, 11.3% male
Morey et al. (2017) [91]	UK	2013	–	2000 (13–15: 54.2%; 16–18: 50.2%)	RE	SH	No	Single-item question	Lifetime: overall 15.5% females 23.1%, males 7.1%
Nguyen et al. (2020) [92]	Vietnam	2018	11	648 (47.7%)	RA	SH	No	Single-item question	Lifetime: 7.1%
Oktan (2017) [93]	Turkey	2016	17.02 (1.59)	263 (54.3%)	C	SHB	Yes	Validate for SIB	Lifetime: 44.86% overall 39.16% females, 51.67% males
Pawłowska et al. (2015) [94]	Poland	–	16.92 (1.15)	6883 (69%)	C	Self-injury	No	Not validate	Lifetime: 24.91% overall 16.24% females, 8.67% males
Pawłowska et al. (2016) [95]	Poland	–	16.91 (1.11)	5685 (30%)	C	Self-injury	No	Not validate	Lifetime: 14% overall 6.92% males, 15.74% females
Peng et al. (2019) [96]	China	2016	13.6 (1.1)	2647 (51.2%)	RE	SH	Yes	Single-item question	6-months:1.4% females, 1.3% males
Pisinger et al. (2018) [97]	Denmark	2014	17.9 (1.5)	66,284 (62%)	RE	SH	No	Single-item question	Lifetime: 20% overall 24% females, 12% males
Plener et al. (2015) [98]	Germany	–	14.85 (0.58)	452 (46.2%)	C	NSSI	Yes	Validate for SIB	Lifetime: 20.4% overall 29.76% females, 12.97% males
Plener et al. (2016) [99]	Germany	2014	15.91	91 (57.1%)	RE	NSSI	Yes	Validate for SIB	Lifetime: 26.9% overall 1.92% males 12.82% females
Quarshie et al. (2020) [100]	Ghana	2017	16.8 (1.38)	444 (51.8%)	RE	SH	No	Single-item question	Lifetime prevalence: 23.8% males, 30% females; 1-year prevalence: 24.8% females, 19.2% males
Reigstad and Kvernmo (2017) [101]	Norway	2003–2005	–	4881 (50.1%)	C	DSH	No	Single-item question	1-year: 22.3% overall 28.8% females, 15.9% males
Ren et al. (2018) [102]	Taiwan	–	15.45 (0.54)	1989 (52.0%)	RE	NSSI	Yes	Validate for SIB	1 year: 20.8% overall 24.4% females, 16.8% males
Schwartz-Mette and Lawrence (2019) [103]	USA	2016–2018	15.68 (1.49)	186 (69.9%)	C	NSSI	Yes	Single-item question	1 year: 27.4% overall 21.43% males, 30% females
Sigurdson et al. (2018) [104]	Norway	1998, 1999–2000	BL: 13.7 (0.58); FU: 14.9 (0.6)	BL: 2464 (50.8%); FU1: 2432 (50.4%)	RE	SH	No	Single-item question	Lifetime: BL—2.48% males, 7.19% females; FU—4.89% males, 11.58% females
Simioni et al. (2017) [105]	Brazil	2010–2011	–	2508 (47.2%)	RE	DSH	No	Diagnostic interview	Lifetime: 1.5%
Solis-Bravo et al. (2019) [106]	Mexico	2016	12.3 (1.3)	438 (57.2%)	C	NSSI	Yes	Validate for SIB	Lifetime: 11.5%
Somer et al. (2015) [107]	Turkey	2010–2011	16.8 (1.26)	1656 (55%)	RE	NSSI	Yes	Validate for SIB	Lifetime: 31.3% overall 33% female, 29.4% male
Stanford et al. (2017) [108]	Australia	2014–2015	14.9 (1.6)	1521 (56.4%)	C	SH	No	Single-item question	6-months: 16.8% overall 12.1% male, 20.5% female
Sutin et al. (2018) [109]	Australia	2014	14.4 (0.49)	2948 (48.3%)	RE	SH	No	Single-item question	1-year: 8.8% overall 3.68% males, 14.52% females
Tang et al. (2016) [110]	China	2013–2014	14.7 (1.9)	4405 (49.67%)	RA	NSSI	Yes	Validate for SIB	1-year: 29.2% overall 30.9% females, 27.4% males
Tang et al. (2018) [111]	China	2014–2015	15.2 (1.8)	15,623 (48.5%)	RE	NSSI	Yes	Validate for SIB	1-year: 29% overall 27.94% males, 30.50% females
Tanner et al. (2016) [112]	Australia	2010	14.20 (1.03)	2637 (58.8%)	C	NSSI	Yes	Validate for SIB	Lifetime: 7.2% males, 11.93% females
Tilton-Weaver et al. (2019) [113]	Sweden	2013–2014	13.65 (0.64)	2769 (47.3%)	C	NSSI	Yes	Validate for SIB	6-months: 5%
Tseng and Yang (2015) [114]	Taiwan	–	–	391 (54.73%)	C	NSSI	Yes	Diagnostic interview	1-year: 9.7% overall 18.7% females; 10.2% males
Victor et al. (2018) [13]	USA	2000–2014	13	2127	C	NSSI	Yes	Diagnostic interview	Lifetime: 3%;
Wan et al. (2015) [115]	China	2008	16.1 (2.8)	17,622 (51.2%)	C	NSSI	Yes	Single-item question	Lifetime: 17.0% overall 16.9% males, 17.1% females
Wan et al. (2019) [116]	China	2013–2014	15.44 (1.8)	14,820 (50.2%)	RE	NSSI	Yes	Single-item question	1-year: 26.1% overall 24.3% female, 27.9% male
Wan et al. (2020) [117]	China	2013–2014	15.59 (1.80)	9704 (52.60%)	C	NSSI	Yes	Single-item question	1-year: 38.54% overall 37.11% female, 40.13% male
Wang et al. (2016) [118]	China	–	14.63 (1.25)	5423 (52.6%)	C	NSSI	Yes	Single-item question	6-months: 18.3% overall 21.2% female 14.6% male
Zetterqvist (2016) [119]	Sweden	2011	16.56	3060 (50.5%)	RE	NSSI/NSSI-D	Yes	Validate for SIB	1-year: NSSI at least one episode: 35.1% overall 10.61% female 11.62% male NSSI-D: 6% overall 9.97% female 2.11% male
Zhang et al. (2016) [120]	China	2013–2014	15.18 (1.79)	25,378 (51.4%)	C	NSSI	Yes	Single-item question	Lifetime: 27.5% overall 28.6% male, 26.4% female
Zubrick et al. (2015) [121]	Australia	2013–2014	15.51 (1.75)	2653 (48.4%)	RE	SH	Yes	Single-item question	Lifetime: 10.9% overall 7.45% males, 17.68% females 1-year: 8% overall 4.6% males, 11.99% females

*SD *standard deviation, *C* convenience, *RA* randomized, *RE* representative, *SITB* self-injurious thoughts and behavior, *SIB* self-injurious behavior, *NSSI* nonsuicidal self-injury, *NSSI-D* nonsuicidal self-injury based on the Diagnostic and Statistical Manual of Mental Disorders 5th Edition criteria, *D-SIB* deliberate self-injurious behavior, *SH* self-harm, *DSH* deliberate self-harm, *SHB* self-harm behavior, *BL* baseline, *FU* follow-up

When multiple studies reported on the same database, we included the ones with the largest sample size [13], the ones that provided data separately for males and females [14], and the ones that provided follow-up results [15, 16]. This led to the exclusion of six studies [17–22]. In addition, we contacted by email the authors of articles from which prevalence data could not be extracted. In case we did not receive sufficient statistics, we excluded the study (e.g., Carvalho et al., 2015). The methodology of this review follows the PRISMA guidelines [23].

### Data extraction

Two authors (BFF, NK) coded the following information:

1. bibliographic information: authors, year of publication and data collection;
2. sample information: age range and mean age of sample, gender ratio, country, and continent the sample was recruited in, representativeness of the community sample, design;
3. measurement of SIB: measurement instrument, suicidal intent, terminology;
4. information for effect size: prevalence estimate and sample size.

Interrater reliability ranged from 73 and 100%. In case a consensus could not be reached between the two coders, the other two authors were consulted (ZKT, JB).

To test our hypotheses, we preferred to include the prevalence estimates separately for males and females if a study reported on those. For longitudinal studies, prevalence at all measurement points was coded; however, they were averaged to calculate an effect size for a study before we included the data in any analyses. We made an exception when prevalence estimates were available separately for males and females at one time point but not at another. In those cases, we chose to include only the estimates at the time point when they were reported separately for males and females.

During the coding, we had to impute some scores that were not reported in the primary studies. For studies that reported only the age range, we imputed the mean age as the geometric mean of the range. For studies that did not report the year of data collection, we subtracted 2 years from the year of publication (for a similar procedure, see Protzko et al., 2020) [24].

### Statistical analyses

We used the Comprehensive Meta-Analysis software to conduct the analyses [25, 26]. We applied a random effects model. When a study reported results at more than one time point, we entered all in the software, which takes the average between multiple time points before entering a study in the grand average. We made an exception when conducting meta-regression analyses regarding the year of data collection and the mean age of the sample. In these cases, we only selected the first time point from these longitudinal studies to be included. In contrast, we considered estimates for males and females when reported separately in a study as independent effect sizes in all the analyses. Outliers were inspected based on a standardized residual exceeding ±3.29. We inspected the results according to several moderator variables. When inspecting results according to the different continents and suicidal intent, we conducted a subgroup analysis to statistically contrast them. We only included subgroups with at least four effect sizes in this analysis (for a similar procedure see Takacs and Kassai 2019) [27].

## Results

### Included studies

**Fig. 1:**
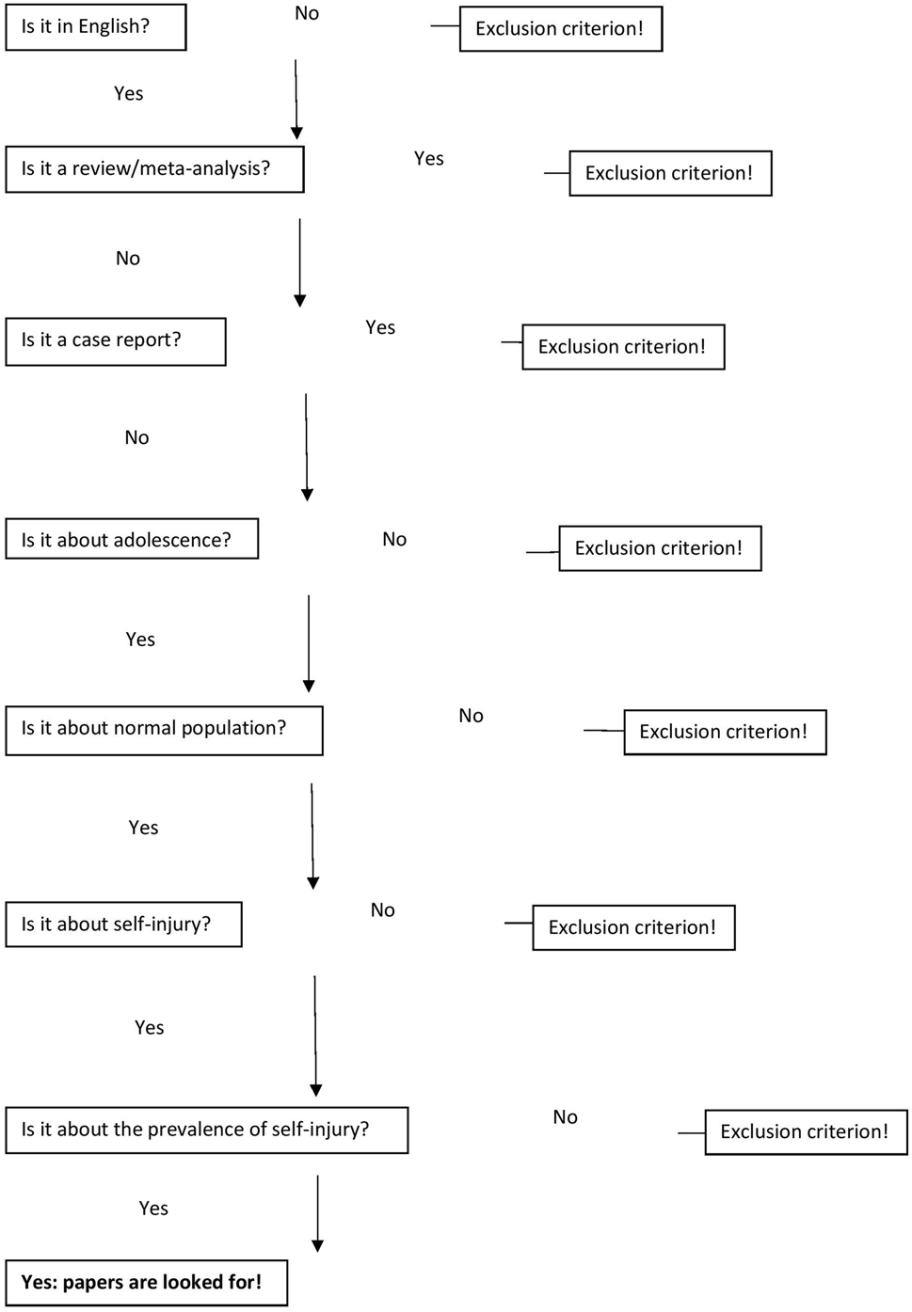
The selection process is summarized in the QUORUM flowchart

**Fig. 2:**
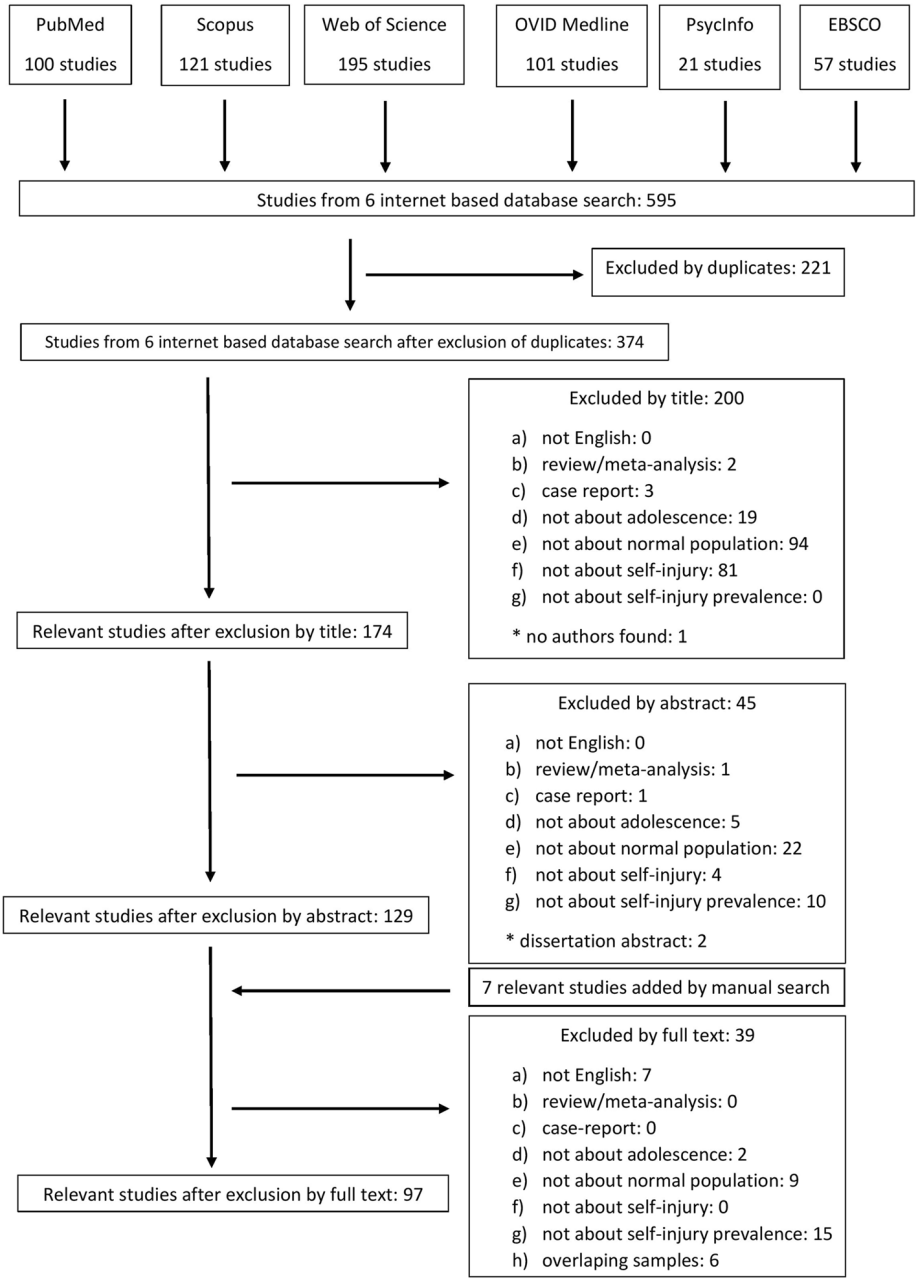
The flowchart of inclusion and exclusion criteria

In sum, a total of 97 articles were included in this meta-analysis; we identified 178 effect sizes (see Figs 1, 2 and Table 2).

There were six outlying effect sizes that we excluded. Altogether, we had data from 439,818 participants. The overall average SIB prevalence in the studies was 16.0% (95% confidence interval [CI] [14.7, 17.4], *k* = 172). This was a heterogeneous effect, *Q*(171) = 30,136.96,* p* < 0.001, *I*^2^ = 99.43 τ^2^ = 0.44.

In our assessment of publication bias, Egger’s test showed significant asymmetry (intercept = −2.88, *p* = 0.046), but the funnel plot showed a symmetric distribution based on visual inspection, which was confirmed by no imputed studies in the Duval and Tweedie’s trim-and-fill procedure.

Among the 97 included articles, 74 reported prevalence data for females and males separately. There were 79 effect sizes reported for females. Two effect sizes were outliers and thus were excluded. We found an average prevalence of 19.4% for females, 95% CI [17.5, 21.4], *k* = 77. This effect was heterogeneous, *Q*(76) = 8,660.74, *p* < 0.001, *I*^2^ = 99.12 τ^2^ = 0.29. There were 75 effect sizes reported for males. One outlying effect size was excluded. We found an average prevalence of 12.9%, 95% CI [11.3, 14.8], *k* = 74. Again, this was a heterogeneous effect, *Q*(74) = 10,315.75, *p* < 0.001, *I*^2^ = 99.2 τ^2^ = 0.43.

### Terms and definitions of SIB in the included studies

The terminology of SIB was not uniform across the included studies. All the studies defined SIB as a deliberate damage to oneself, but not all of them defined it as a nonsuicidal intent. Seventy-two articles (73.5%) made a clear distinction between suicidal and nonsuicidal intent.

There were 11 different terms for SIB in the included 97 papers. The most frequently used term was NSSI; this expression appeared in 60 articles (see Table 3).

**Table 3:** Prevalence differences in the terms of SIB

	Prevalence estimates (95% CI)		
	Overall	Only female samples	Only male samples
DSH	15.1% (11.2–20.2) (k = 8)	11.5% (6.2–20.4) (k = 4)	6.3% (3.2–11.8) (k = 4)
Deliberate self-injurious behavior (D-SIB)	16.2% (9.7–25.8) (k = 12)	20.1% (10.8–34.4) (k = 6)	12.8% (7.3–21.5) (k = 6)
Non-fatal self-harm	4.9% (1.5–15.3) (k = 2)	8.9% (7.9–10) (k = 1)	2.7% (2.1–3.3) (k = 1)
NSSI	18.4% (16.9–20) (k = 103)	20.8% (18.2–23.7) (k = 47)	17.1% (15.1–19.3) (k = 44)
NSSI based on the Diagnostic and Statistical Manual of Mental Disorders 5th Edition (DSM-5) criteria	5.3% (2.6–10.6) (k = 4)	10.6% (8.8–12.8) (k = 2)	2.5% (1.8–3.4) (k = 2)
Self-cutting	7.4% (4.8–11.2) (k = 4)	10.4% (8.2–13.2) (k = 2)	4.9% (4–6) (k = 2)
Self-injury	12.4% (8.2–18.4) (k = 5)	16.1% (15.2–17.1) (k = 2)	7.7% (6.2–9.6) (k = 2)
Self-harm (SH)	12.7% (10–16) (k = 34)	18.2% (13.5–24.1) (k = 13)	9% (5.5–14.4) (k = 13)
Self-harm behavior (SHB)	45.2% (33.4–57.6) (k = 2)	39.2% (31.5–47.4) (k = 1)	51.7% (42.8–60.5) (k = 1)
SIB	18.3% (10.2–30.6) (k = 2)	24% (21.4–26.8) (k = 1)	13.7% (11.9–15.7) (k = 1)
Self-injurious thoughts and behavior (SITB)	14.4% (9–22.2) (k = 2)	16.1% (8.9–27.4) (k = 1)	12% (5.5–24.2) (k = 1)

### Measurements of SIB in the included studies

Among the included studies we found diagnostic interviews, self-reported questionnaires, and single-item questions to measure SIB. Two studies measured NSSI based on DSM-5 criteria [122]. The most frequently used questionnaire was the Deliberate Self-Harm Inventory [123], which was mentioned in 13 articles. The Inventory of Statements About Self-Injury [124] was used in five studies, and the Functional Assessment of Self-Mutilation [125] also was used in five. Effect sizes based on a single item to assess SIB found an average prevalence of 11.6%, 95% CI [9.3, 14.5], *k* = 31. We found of 14.8%, 95% CI [12.8, 17.2], *k* = 60, in studies that used nonvalidated questionnaires. Questionnaires that had been validated for other constructs showed an average prevalence of 14.7%, 95% CI [9.8, 21.5], *k* = 6. Finally, questionnaires that had been validated for SIB showed the highest average percentage: 18.9%, 95% CI [16.9, 21.1], *k* = 77. For results separately for males and females, see the Supplementary Materials. Only one study used a diagnostic interview and reported on two effect sizes. The average of these showed a similar estimate as the grand average (14.2% (95% CI [7.7, 24.8], *k* = 2), more specifically, 18.7% for females and 10.2% for males).

### Sampling

Of the 172 effect sizes, 99 were based on convenience sampling. These showed an average prevalence of 15.2%, 95% CI [13.4, 17.2]. Eighteen effect sizes were based on samples that applied randomization, showing a prevalence of 24.7%, 95% CI [18.9, 31.6]. For 55 sample sizes, the sample was representative of the population. Representative samples showed a pooled prevalence of 15.1%, 95% CI [13.2, 17.4]. A similar pattern was noted for females and males (see the Supplementary Materials).

### Place of data collection of the included studies

From the 98 included articles, we found three collaborations in which data were collected in multiple countries; for the rest, the data were collected in single countries. When we inspected the results over all the effect sizes, we noted differences according to the continent on which the data had been collected. There were three effect sizes in two publications from South America that showed an average prevalence of 33%, 95% CI [13.7, 60.3], and we found two effect sizes in one publication from Africa that showed an average prevalence of 24.4%, 95% CI [19.1, 30.7], and data for two effect sizes published in the same article were collected in North America and Australia and Oceania as part of an international cooperation that showed a prevalence of 2.6%, 95% CI [1.3, 4.9]. These categories were excluded from the subgroup analysis as they contained less than 4 effect sizes. After we excluded these, we noted a significant difference between the prevalence estimates from the different continents (see Table 4), *Q*(3) = 10.97, *p* = 0.012. More specifically, prevalence estimates from Asia (19.5%, 95% CI [17.1, 22.2], *k* = 51) were significantly larger than those from the other three continents (14.6%, 95% CI [13.1, 16.2], *k* = 114), *Q*(1) = 11.20, *p* = 0.001. As shown in Table 4, the effect of continent was similar when we inspected effect sizes for female and male samples separately.

**Table 4:** Prevalence differences in continental distribution

	Prevalence estimates (95% CI)		
	Overall	Only female samples	Only male samples
Asia	19.5% (17.1–22.2) (k = 51)	22.3% (19.4–25.4) (k = 23)	19.5% (16.6–22.8) (k = 22)
Australia and Oceania	14.1% (9.5–20.5) (k = 13)	18.5% (11.8–27.8) (k = 6)	10.0% (4.3–21.5) (k = 6)
Europe	14.7% (12.9–16.8) (k = 87)	19.5% (17.0–22.4) (k = 37)	10.8% (8.5–13.79) (k = 36)
North America	13.8% (10.2–18.4) (k = 14)	14.3% (10.5–19.2) (k = 7)	11.5% (5.9–21.1) (k = 6)

### Mean age of the included samples

For assessing the effects of the mean age of the samples, we chose to focus on the first measurement point in the 17 longitudinal studies. In this analysis, seven outliers appeared that were then excluded. For an additional six effect sizes we could not extract the sample’s age, and thus those were also excluded from this analysis. This resulted in 165 effect sizes. The mean age of the sample ranged from 11.00 to 18.53 years. The mean age of the sample did not have a significant effect on the effect size (coefficient = 0.067, *p* = 0.12). For results separately for males and females, see the Supplementary Materials.

To make sure that longitudinal studies from which we chose to include the first estimate in this analysis did not influence the results by possibly reporting on substantially younger samples, we also ran the regression model on the cross-sectional studies only as a sensitivity analysis. This resulted in 134 effect sizes to be included. Again, the mean age of the sample did not have a significant effect on these prevalence estimates (coefficient = 0.058, *p* = 0.24).

### Suicidal intent

For 125 effect sizes, suicidal intent was excluded. Those showed a pooled estimate of 18.3%, 95% CI [16.7, 19.9]. This was significantly higher than what was found in studies that did not exclude suicidal intent (11.3%, 95% CI [9.3, 13.7], *k* = 47), *Q*(1) = 20.52, *p* < 0.001. This pattern was also confirmed in only-female and only-male samples. For results separately for males and females, see the Supplementary Materials.

### Prevalence of SIB

We found 92 effect sizes reporting on lifetime prevalence of SIB, 72 effect sizes that estimated 1-year prevalence, and 17 that estimated 6-month prevalence. An average of 17.9%, 95% CI [16.3, 19.5], was found overall when lifetime prevalence was assessed. This estimate was 22.9 (95% CI [20.9, 25.0], *k* = 42) for females and 13.7% (95% CI [11.2, 16.8], *k* = 39) for males.

An overall average prevalence of 13.4%, 95% CI [11.5, 15.6] was found when assessing prevalence in the last year. This estimate was 15.9% (95% CI [12.9, 19.4], *k* = 32) for females and 10.7% (95% CI [8.7, 13.2], *k* = 32) for males. An overall prevalence of 16.2%, 95% CI [11.0, 23.3] was estimated when we considered only the last 6 months, 18% (95% CI [8.9, 33.2], *k* = 7) for females and 13.8% (95% CI [6.2, 27.9], *k* = 7) for males.

### Year of data collection

In regard to assessing the effects of the year of data collection, we chose to focus on the first measurement point in the 17 longitudinal studies. In this analysis, seven outliers appeared that were then excluded. Data for the primary studies were collected between 1998 and 2018. The year of data collection had a significant, positive effect on the 171 effect sizes (coefficient = 0.035, *p* = 0.008); that is, more recent studies found larger prevalence. For results presented separately for females and males, see the Supplementary Materials.

For further investigation, we restricted the year of data collection to 2013 and onward so we could assess the effect in the time constraints that corresponds to the time constraints of year of publication of the present meta-analysis (2015 and onward). Data for 119 effect sizes were collected in or after 2013. When we considered only these studies, the effect of year of data collection was not significant on the effect sizes (coefficient = −0.015, *p* = 0.72). The same was found for the 53 effect sizes for females (coefficient = −0.005, *p* = 0.92) and for the 51 effect sizes for males (coefficient = −0.05, *p* = 0.49). Scatterplots are shown in the Supplementary Materials.

### Risk of bias

Risk-of-bias criteria was based on the Cochrane Risk of Bias Tool [126], adapted for the studies (cohort, cross-sectional, and longitudinal).

## Discussion

Because previous meta-analyses have yielded conflicting results on the prevalence of SIB in community adolescent samples [6, 11], we found it important to complete a follow-up meta-analyses with clear methodology on recently published data. In the present meta-analysis, we found that the prevalence of SIB in adolescents was 16% in studies published between 2015 and 2020. This result is comparable to the estimate of 16.9% found in a previous meta-analysis [6]. Regarding methodological differences, as can be expected, a slightly higher estimate was found when considering lifetime prevalence (17.9%) as compared with the 1-year (13.4%) or 6-month prevalence (16.2%). We also noted a significantly higher prevalence when suicidal intent was excluded (18.3%) than when it was not excluded (11.3%), and the largest prevalence was found when measurement instruments were used that had been validated for self-injurious behaviors (18.8%). In addition, methodologically more rigorous studies that focused on representative samples found an average SIB prevalence of 15.1%. This is an interesting issue, while self-harm without suicidal intent should be a subgroup of self-harm covering forms both with and without suicidal intent. Hence the first number should always be lower than the second number. A possible explanation could be that the studies used the same term but actually employ different criteria. In addition, differences in the prevalence of NSSI and DSH may also result from measurement differences between the two types of SIB. Previous meta-analyses have reported higher prevalence rates for multi-item instruments [6, 10], and 65.5% of NSSI measurements consisted of multiple items, compared to 60% of DSH measurements consisting of a single item. In a meta-analysis made by Swannell et al. (2014), checklist versus single-item measurement explained the 41% of variance between studies [9]. Our review shows that, among adolescents, there are no significant changes in the prevalence between ages 11.0 and 18.5 years. This result is comparable to Lim’s meta-analysis [12] but does not align with Gillies and colleagues’ (2018) study. We found similar prevalence estimates among studies that used convenience and representative samples. However, and surprisingly, studies that used a random sample found larger estimates. This is puzzling and needs further research.

Our first hypothesis was only partially confirmed. When we considered all data that were published between 2015 and 2018, we found that there was a significant increase between 1998 and 2018 in the prevalence of SIB. However, when we restricted our analysis to the time frame between 2013 and 2018 (to reflect the publication time window of 2015 and 2018), we found no change in prevalence, as we had expected. Previous meta-analyses have found mixed results regarding this question. Muehlenkamp and colleagues (2012) did not find any significant difference in the prevalence of SIB between 2008 and 2015, whereas Gillies and colleagues (2018) found an increase between 1990 and 2018. Our results are in line with both previous findings in that they show an increase before 2013, but no change since then. This finding can be important to both decision makers and professionals for the appropriate planning of prevention programs.

Our second hypothesis was confirmed; we found a substantial difference between the estimates for females (19.4%) and males (12.9%), with nonoverlapping confidence intervals. A similar pattern was observed when we considered only lifetime prevalence, with 22.9% for females and 13.7% for males. These results are comparable to Bresin and Schoenleber’s (2015) meta-analysis, in which the prevalence was significantly higher among females. Studies that excluded suicidal intent found an average of 21% for females and 16.5% for males. Similarly, estimates based on measurement instruments that were validated for SIB showed 21.9% for females and 15.7% for males. On the other hand, studies with representative samples showed slightly lower estimates: 18.2% for females and 10.9% for males. Thus, the patterns were very similar for females and males when the effect of methodological differences in the primary studies were assessed. However, the cultural difference between countries in Asia and those on other continents was more articulated for males (Asia = 19.5% vs. other = 10.8%) than for females (Asia = 22.3% vs. other = 18.5%). Nock and Prinstein (2005) found that NSSI often is connected to psychological distress [127], and adolescent girls usually have more psychological distress than men [128]. These results highlight that it is necessary to pay more careful attention to NSSI by female adolescents and that perhaps further attention should be given to Asian male populations.

We found some differences in the prevalence estimate as a function of methodological differences among the primary studies; however, we should note that moderators might be confounded.

The pooled estimate from Asian countries (19.5%) was significantly higher than that from other continents (14.6%). Again, this confirms earlier meta-analytic results estimating a relatively large prevalence in Asian countries (Lim et al. 2019). This difference was even more articulated for males. So, it may be that the differences in SIB between Asian and non-Asian countries are somehow connected to gender. To understand this result, further research should focus on the transcultural aspects of SIB.

We did not find a difference between NSSI (18.7%) and DSH (15.1%), unlike Gillies’s results [6], but we found a substantial difference between NSSI (18.7%) and self-harm (12.7%). In contrast to females, we found a substantial difference between NSSI (17.1%) and DSH (6.3%), and between NSSI (17.1%) and self-harm (9%) among males.

Our review highlights that the highest prevalence rates were found when SIB was measured with a validated questionnaire as compared with studies that used single-item or nonvalidated questionnaires, a pattern that was also confirmed separately for male and female samples. This result is likely due to the fact that validated questionnaires are more sensitive than single-item measures [6, 10].

Our results are limited by the heterogeneity of the primary studies, that is, in regard to the sample and the measurement instruments and the conceptualization of SIBs. The findings of the present meta-analysis confirm that these differences among the primary studies have an important effect on the prevalence estimates. There is currently no consensus in the literature about the conceptualization of SIB [6, 10], which makes our work more difficult when evaluating the data. However, to provide the most precise estimate, we pooled the studies that used representative samples that reported on lifetime prevalence of SIBs excluding suicidal intent measured by a validated measurement instrument and found similar estimates. In addition, we did this to avoid a confound effect of these moderators. Moreover, although overall we found a relatively large number of studies that reported on prevalence of SIBs, it is questionable whether nonsignificant results in subgroup and meta-regression analyses are truly due to an absence of an effect or whether they are instead due to a lack of statistical power.

To our best knowledge, this is the most recent meta-analysis on the prevalence of SIB among adolescents. An overall prevalence of 16% was found, which means that one in six adolescents has a history of self-harm. Moreover, a larger estimate was found for females as compared with males: every fifth adolescent girl reported having conducted self-harm. It is interesting that estimates were largest in Asian countries with males, approaching a 20% prevalence. Further research should focus on the transcultural aspects of self-harm to understand this difference. All these results have public health importance in drawing the attention of clinicians and decision makers to adolescents who engage in SIB. Clinicians need to be aware of the high prevalence and risk factors (e.g., female gender, Asian populations) of SIB in adolescence. Prevention and intervention are very important in this age group.

## Supplementary Information

Below is the link to the electronic supplementary material.

Supplementary file1 (DOCX 13 KB)

## Data Availability

Not applicable.

## Abbreviations

DSH: Deliberate self-harm
D-SIB: Deliberate self-injurious behavior
DSM-5: Diagnostic and statistical manual of mental disorders 5th edition
NSSI: Nonsuicidal self-injury
SH: Self-harm
SHB: Self-harm behavior
SIB: Self-injurious behavior
SITB: Self-injurious thoughts and behavior
