# Supplementary material for: The prevalence of self-injury in adolescence: a systematic review and meta-analysis
Source: Eur Child Adolesc Psychiatry. 2023 Jul 24;33(10):3439–58. doi: 10.1007/s00787-023-02264-y (PMC11564408; doi:10.1007/s00787-023-02264-y)
Supplement: Supplementary file 1 — Supplementary file1 (DOCX 13 KB) [file 787_2023_2264_MOESM1_ESM.docx]

Supplementary materials:

**Moderator analyses separately for males and females**

**Measurement of SIB**

The pattern found overall was confirmed when considering females samples (single item: 16.5% (95% CI = 11.9 – 22.4, k = 13), non-validated questionnaires: 17.2% (95% CI = 15 – 19.6, k = 29), questionnaires validated for other than SIB: 20.4% (95% CI = 13.5 – 29.7, k = 2), questionnaires validated for SIB: 21.9% (95% CI = 18.4 – 25.9, k = 35). The pattern was only partially confirmed for males (single item: 8.6% (95% CI = 5.3 – 13.6, k = 13), non-validated questionnaires: 11.7% (95% CI = 9.2 – 14.9, k = 29), questionnaires validated for other than SIB: 19% (95% CI = 8.3 – 37.8, k = 2), questionnaires validated for SIB: 15.7% (95% CI = 12.7 – 19.2, k = 32).

**Sampling**

For females, estimates were similar when convenience (18.5%, 95% CI = 16.1 – 21.1, k = 43) and representative samples (18.2%, 95% CI = 15.4 – 21.4, k = 26) were used, while effect sizes based on random samples showed a pooled prevalence of 29.8% (95% CI = 20.0 – 42.0, k = 8). For males, convenience samples showed an average prevalence of 12.8% (95% CI = 10.4 – 15.7, k = 40), random samples showed 22.3% (95% CI = 13.9 – 33.9, k = 8), while representative samples found 10.9% (95% CI = 8.7 – 13.6, k = 26).

**Year of data collection**

For females, three outliers were excluded resulting in 76 effect sizes for this analysis. The year of data collection had a tendency-level effect on the effect sizes (coefficient: 0.031, *p* = .06). For males, two outliers were excluded resulting in 73 effect sizes. For males year of data collection had a significant positive effect (coefficient: 0.054, *p* = .007), again, suggesting larger prevalence in more recent studies.

**Suicidal intent**

For females, studies that excluded suicidal intent found a significantly higher prevalence (21%, 95% CI = 18.4 – 24, k = 56) than studies that did not (15.5%, 95% CI = 13.2 – 18.2, k = 21), *Q*(1) = 7.95, *p* = .005. Similarly, for males, studies that excluded suicidal intent found a significantly higher prevalence (16.5%, 95% CI = 14.6 – 18.6, k = 53) than studies that did not (7.1%, 95% CI = 4.8 – 10.4, k = 21), *Q*(1) = 17.31, *p* < .001.

**Mean age of the sample**

For the 74 effect sizes reported for females, the mean age of the sample did not have an effect (coefficient: 0.04, *p* = .55). Nor did it for the 71 effect sizes for males (coefficient: 0.09, *p* = .23).
